# Complete and Removable Partial Prosthesis for a Child with Hypohidrotic Ectodermal Dysplasia

**DOI:** 10.5005/jp-journals-10005-1192

**Published:** 2013-04-26

**Authors:** Bhavesh D Trivedi, Rupinder Bhatia

**Affiliations:** Postgraduate Student, Department of Pediatric and Preventive Dentistry Padmashree Dr DY Patil Dental College and Hospital, Navi Mumbai Maharashtra; Professor and Head, Department of Pediatric and Preventive Dentistry Padmashree Dr DY Patil Dental College and Hospital, Vidyanagar, Nerul Navi Mumbai, Maharashtra, India

**Keywords:** Hypodontia, Hypohidrotic ectodermal dysplasia, Complete denture, Removable partial denture, Esthetic and functionality

## Abstract

Young children with hypodontia caused by hypohidrotic ectodermal dysplasia (HED) not only have difficulties in mastication and speech but can also sense that their appearance is different from others. Enabling children with HED to look like their peers through the use of well-fitting and functioning complete and removable partial dentures with age appropriate teeth will greatly assist in their transitioning in to their school years and add to their psychological well-being. Although denture construction requires multiple patient appointments and good co-operation, dentist also should educate and encourage parents and patient to tackle the difficulties that may come across during denture construction. In this present case the child, a 4-year-old, required a complete denture in the lower arch and removable partial denture in upper arch to achieve functionality and enhanced esthetics.

**How to cite this article:** Trivedi BD, Bhatia R. Complete and Removable Partial Prosthesis for a Child with Hypohidrotic Ectodermal Dysplasia. Int J Clin Pediatr Dent 2013;6(1):71-74.

## INTRODUCTION

Ectodermal dysplasia is the term used to describe a group of rare, inherited disorder characterized by dysplasia of tissues of ectodermal origin primarily nail, skin and teeth and occasionally, dysplasia of mesodermally derived tissues.^1^ The condition is thought to occur in approximately 1:10,000 to 1:1,00,000 live births and is more frequent in males. The majority of cases follow the autosomal recessive mode of inheritance but it can also be autosomal-dominant or X-linked.^2^The most common type observed is the hypohidrotic-anhidrotic and the hidrotic. The hypohidrotic-anhidrotic type or Christ-Siemens-Touraine syndrome was first described by Thurman in 1848 and is characterized by hypotrichosis (skin, hair and nail anomalies), hypodontia or anodontia and hypohidrosis (partial and total absence of sweat glands) and features such as frontal bossing, saddle nose, everted lips, etc.^3^

The hidrotic type was first defined in 1929 by Clouston and is distinguished by hypotrichosis, ungual dystrophy and hyperkeratosis of the palms and soles.^4^

Numerous combinations of clinical alteration can present in ectodermal dysplasia, observing diverse syndromes and up to 154 different types of ectodermal dysplasia and 11 subgroups, labeled from 1 to 4 according to whether they affect the hair, teeth, nails or sweat glands.^5^

Recently a new classification for ectodermal dysplasia has been proposed, based on the alteration in the protein molecular functions that lie behind it.^6^

The diagnosis of patients with ectodermal dysplasia is based fundamentally on the clinical history (ungual dystrophy, hypotrichosis, anodontia, oligodontia, hypodontia), skin biopsy showing reduction in pilosebaceous units and sweat glands, hair study showing thin, fine hair, panoramic radiography clearly showing dental dysmorphia and agenesis and molecular genetic analysis.^7^

Oral manifestations of hypohidrotic ectodermal dysplasia (HED) are of particular interest to the dentist, because patients with this disorder invariably have missing or malformed teeth. It can affect both the primary and permanent dentition. Because of partial development of teeth or the absence of teeth in patients with HED, the restoration of the dentition to proper form and function can be a significant challenge to the dentist, particularly in a young child.^8^

## CASE REPORT

A 4-year-old female patient visited the Department of Pediatric and Preventive Dentistry of Padmashree Dr DY Patil Dental College, with the complaint of missing teeth in the maxillary arch and complete absence of teeth in the mandibular arch. She had difficulty in mastication and speech. Peers teased her about her appearance which was constant psychological trauma to the patient and parents. Patient had history of absence of sweating even in hot summer, frequent rise of body temperature since early infancy and getting micturition reflex frequently. Family history revealed consanguineous marriage of parents. Parents and other family members were normal.

**Fig. 1:**
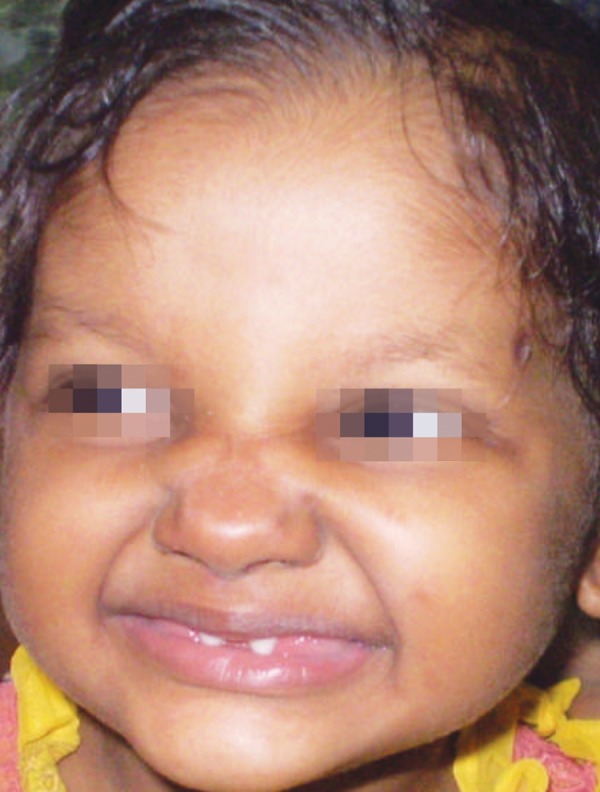
Front view showing classical features of child with ectodermal dysplasia

**Fig. 2:**
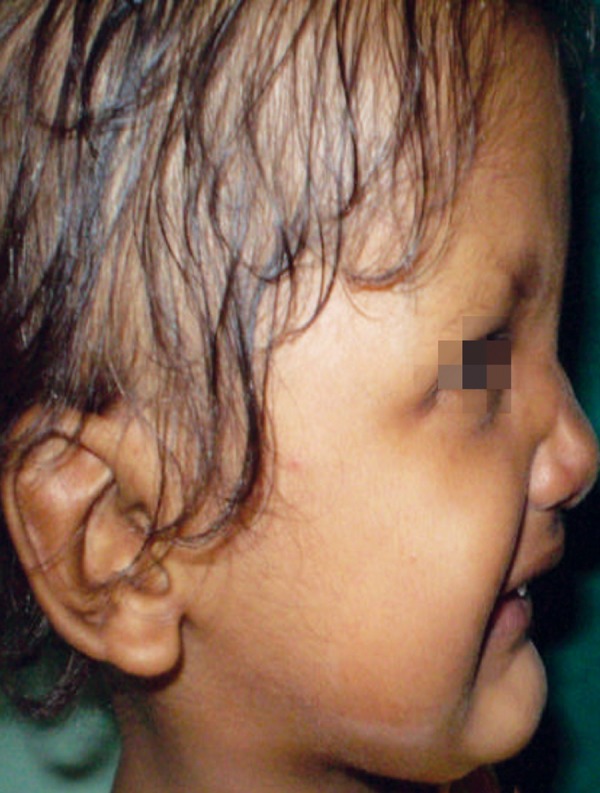
Side view showing classical features of child with ectodermal dysplasia

On extraoral examination, patient exhibited classical features of ectodermal dysplasia. She had fine sparse hair on scalp and lack of hair on rest of the body, prominent forehead, saddle nose, everted lips (Figs 1 and Figs 2).

**Fig. 3:**
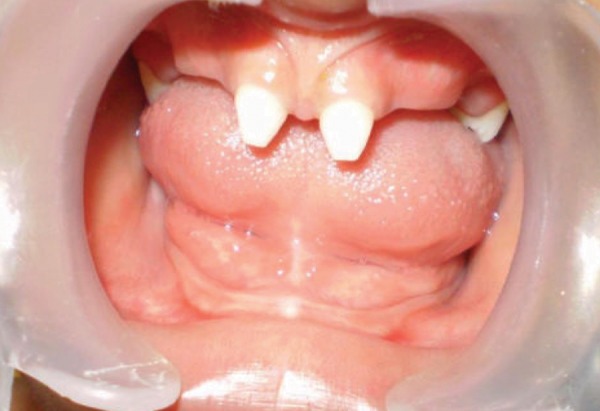
Intraoral view showing partial anodontia of maxillary jaw with complete anodontia of mandibular jaw. Aplasia of alveolar bone in the edentulous areas is clearly seen

Intraoral examination revealed conical-shaped deciduous centrals, right and left second deciduous molars in the upper arch and edentulous lower arch. She exhibited aplasia of alveolar bone in the edentulous areas (Fig. 3).

**Fig. 4:**
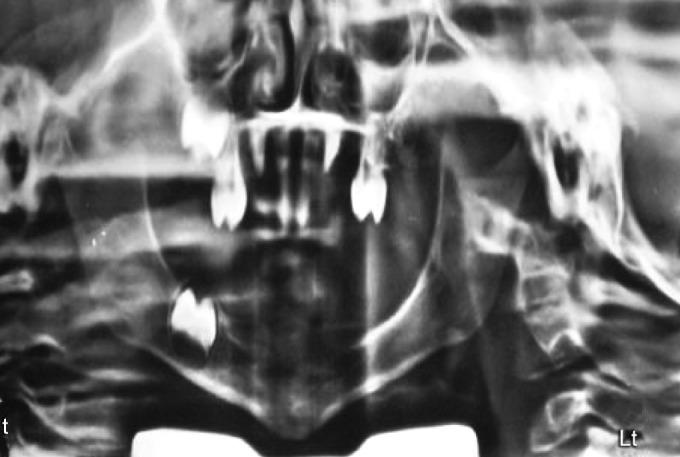
Orthopantogram (OPG) showing developing permanent canines, right permanent first molar in the upper arch and permanent right first molar in the lower arch

*Radiographic examination:* OPG revealed presence of developing permanent canines, right permanent first molar in the upper arch and permanent right first molar in the lower arch (Fig. 4).

**Fig. 5:**
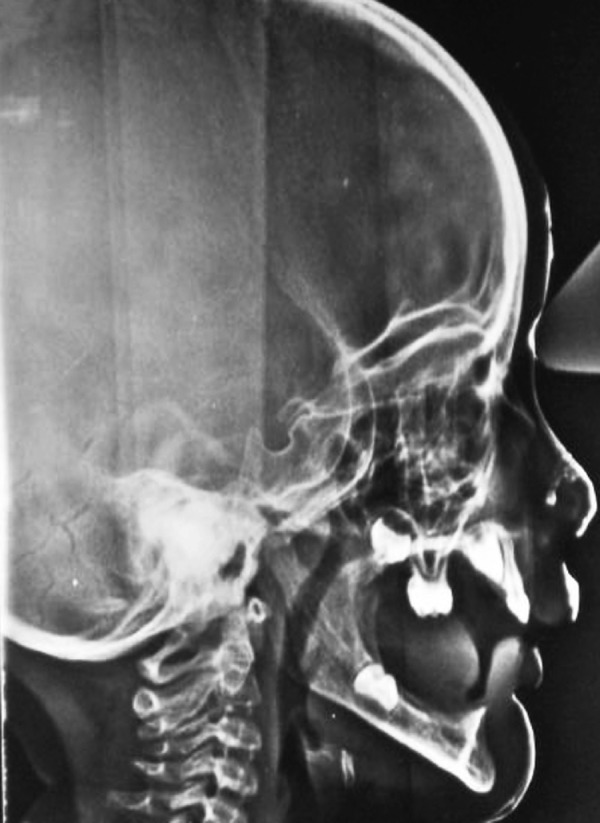
Lateral cephalogram showing aplastic lower jaw

Lateral cephalogram shows aplastic lower jaw (Fig. 5).

Removable partial denture for the upper arch and complete denture for the lower arch to restore function, followed by strip crown with composite restoration for conical-shaped deciduous maxillary centrals to give them proper shape and morphology was planned.

Treatment begun with, making upper and lower primary impression with alginate impression material. Specialized custom-made deciduous acrylic trays fabricated on deciduous ideal cast were used for this purpose. Primary casts were prepared. On lower primary cast special tray was fabricated and final impression was obtained with light-body vinyl siloxane impression material. Master cast was obtained from the final impression. Denture base and occlusal rim was constructed. On the upper cast, direct retainer (c clasp) on both deciduous second molars was made, base plate (shellac base plate) was adapted and occlusal rim was constructed.

**Fig. 6:**
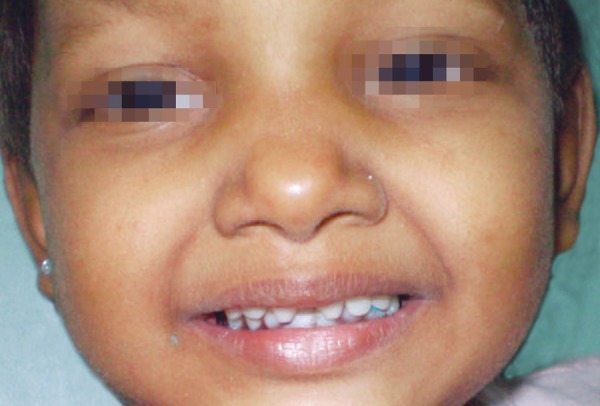
Child patient with trial denture

**Fig. 7:**
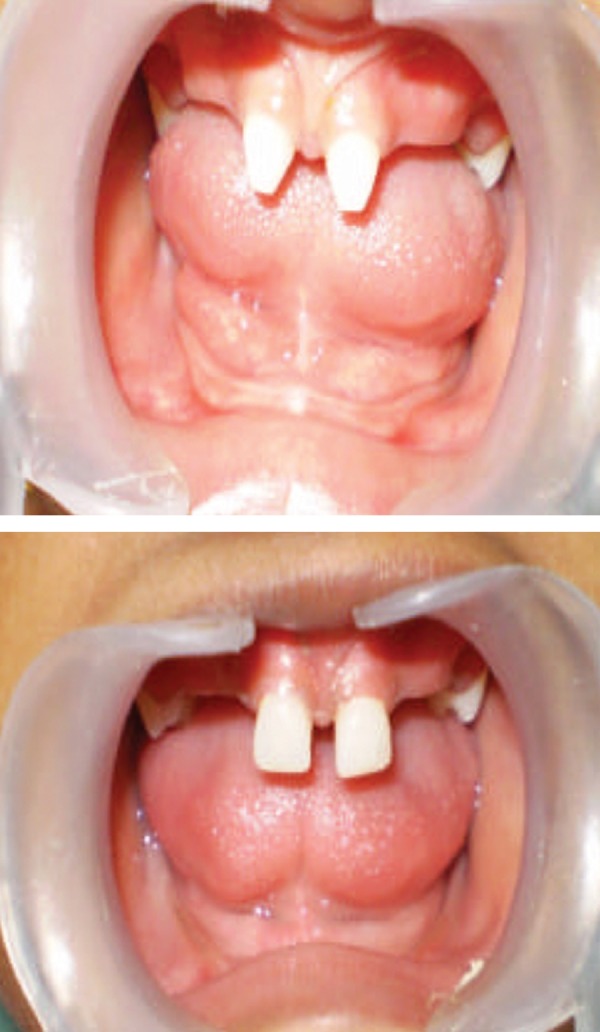
Conical deciduous central incisors modified with strip crowns

Jaw relation was recorded. This recorded jaw relation was transferred on to mean value articulator. Teeth arrangement in lower and upper denture base was done. In the next appointment trial dentures were tried for retention, stability, function and esthetic (Fig. 6). Lower complete denture and upper removable partial denture was constructed with heat cure acrylic by conventional denture making procedure. Morphological modification of two conical-shaped upper deciduous central incisors with composite strip crowns was done to improve the esthetics (Fig. 7). Before delivering the dentures, topical fluoride application was done to maintain the teeth present in the upper arch.

**Fig. 8:**
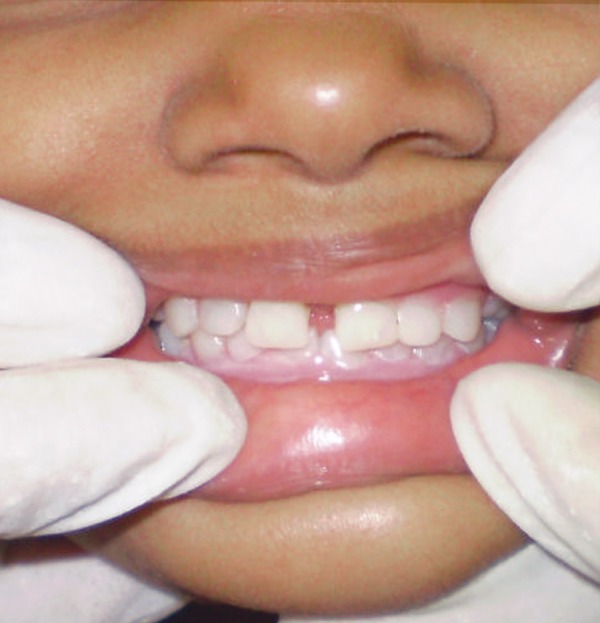
Partial denture for maxillary jaw and complete denture for mandibular jaw

**Fig. 9:**
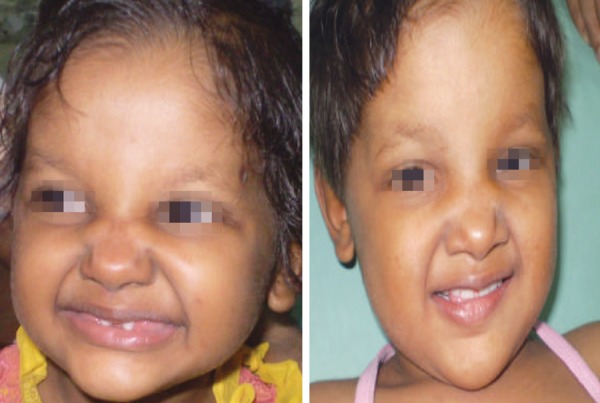
Improvements in facial form and esthetics after placement of prosthesis

Dentures were delivered (Fig. 8). Preoperative and postoperative comparison saw marked improvements in facial form and esthetics (Fig. 9). Instructions regarding maintenance of dentures were given and recall appointments were scheduled after 24 hours, 1 and 3 weeks. Fluoride mouthwash was prescribed. After 3 weeks the patient was well adjusted to the dentures. The parents were very happy and stated that there was a significant improvement in her speech and esthetics, and it has contributed toward her psychological well-being. She was scheduled for recall for every 3 months for evaluating her oral hygiene status and maintenance of dentures.

## DISCUSSION

Nowak stated that ‘treating the pediatric patient with ectodermal dysplasia (ED) requires the clinician to be knowledgeable in growth and development, behavioral management, techniques in the fabrication of a prosthesis, the modification of existing teeth utilizing various restorative techniques, the ability to motivate the patient and parent in the use of the prosthesis, and the long-term follow-up for the modification and/or replacement of the prosthesis'. According to Nowak, a series of introductory visits may be needed before treatment commences, to attain the required patient trust.^9^

A prosthodontic rehabilitation is fundamental in these situations, attempting to provide a functional and esthetic solution that will allow the child as normal a lifestyle as possible, without damaging self-esteem or psychological development and ensuring that behavior is unaffected.^7^

The prosthetic treatment should be carried out on an individual basis, aimed always toward providing good occlusal stability. Treatment should be commenced as soon as possible in order to avoid possible resorption and atrophy of the alveolar ridges, and to control vertical dimension, which can be severely affected by the total or partial lack of teeth.^7^

Different authors have proposed different rehabilitation possibilities for these patients. In general, almost all agree in recommending the use of removable prosthesis during the early stages of growth (3-5 years), allowing the adjustment of the vertical dimension or maxilla/mandible interrelationship. When the patient growth is completed and a more stable and fixed situation is established, possibility of provisional fixed prostheses and of implant treatment can be considered.^4,10-13^

Individual crown restorations have no age restriction related to jaw growth, but larger pulp sizes and shorter crown heights may cause concern. In spite of these concerns, crowns are often used in the treatment of young ED patients.^14^

Recently, direct composite restorations have become the more desirable method of restoring normal morphology to hypoplastic teeth commonly found in ED patients. Crown and direct composite restorations are often used in combination with removable partial dentures in the prosthodontic management of these patients. They are usually necessary to provide proper contours on the hypoplastic and morphologically deformed teeth that will be used as abutments for removable partial dentures. Also, orthodontic treatment may be needed to align the teeth into acceptable positions before removable partial denture fabrication.^15^

Removable prosthesis is the most frequently reported treatment modality for the dental management of young ED patients. Because anodontia or hypodontia is typical in individuals with this condition, complete dentures, partial dentures or overdentures are often parts of the treatment provided. Although complete dentures can provide an acceptable esthetic and functional result, underdevelopment of the edentulous alveolar ridges in individuals with ED can compromise denture retention and stability.^15^

When there are teeth present for support, overdentures is a desirable treatment option for these patients. Cram provided an excellent overview of the advantages of conventional overdentures as opposed to complete dentures. One important advantage is that overdentures preserve alveolar bone as it is imperative in individuals with ED because they must depend on the alveolar ridges for prosthesis support from an early age. Van Wass et al verified this claim with a well-designed, randomized controlled clinical trial where he found there was a significant reduction in alveolar bone loss in the overdenture patients after 2 years. If an overdenture is fabricated, retention can be augmented by various attachments anchored to the available teeth.^15^

Periodic recall of young ED patients is also important because prosthesis modification or replacement will be needed as a result of continuing growth and development. In addition to adjustments related to fit, occlusion of prosthesis must be monitored for ages because of jaw growth. Other problems related to removable prosthesis are speech difficulties, dietary limitations, patient co-operation and loss of the prosthesis.^9^

## CONCLUSION

From our point of view, the use of partial and complete acrylic prostheses is an interesting and practical alternative that provides a relatively quick, easy, acceptable and economical solution to the functional esthetic oral rehabilitation and psychological benefit in young patients with pronounced edentulism. This solution improves the patient's quality of life and optimizes social integration; furthermore, it permits stimulation of the alveolar ridges for later treatment with an implant supported prosthesis as a more stable and esthetic solution for patients with multiple dental agenesis.
